# Biochemical and histopathological effects of copper oxide nanoparticles exposure on the bivalve *Chambardia rubens* (Lamarck, 1819)

**DOI:** 10.1042/BSR20222308

**Published:** 2023-05-18

**Authors:** Mostafa Morad, Taha F. Hassanein, Manal F. El-khadragy, Alaa Fehaid, Ola A. Habotta, Ahmed Abdel Moneim

**Affiliations:** 1 Department of Zoology and Entomology, Faculty of Science, Helwan University, Cairo 11795, Egypt; 2 Department of Chemistry, Faculty of Science, Helwan University, Cairo 11795, Egypt; 3 Department of Biology, College of Science, Princess Nourah bint Abdulrahman University, P.O. Box 84428, Riyadh 11671, Saudi Arabia; 4 Department of Forensic Medicine and Toxicology, Faculty of Veterinary Medicine, Mansoura University, Dakahlia, Egypt

**Keywords:** bivalves, CuONPs, digestive gland, gills, oxidative stress

## Abstract

Copper nanoparticles are widely incorporated into many applications, including air and liquid filters, wood preservatives, batteries, thermal and electrical conductivity, inks and skin products. Their potential toxicity and environmental fate, however, are poorly studied in the freshwater bivalves. The aim of the present study was to evaluate the different effects of copper oxide nanoparticles and ionic copper on the digestive glands and gills of the mussel *Chambardia rubens*. Mussels were treated with 100 and 1000 µg Cu L^−1^ of copper oxide nanoparticles (CuONPs) or ionic copper (Cu^2+^) for 3, 7, and 14 days. The Cu accumulation and markers of oxidative stress in the digestive glands and gills were evaluated. The results show that the digestive gland collected higher levels of the two forms of copper than the gills. Exposure to CuONPs or Cu^2+^ induced significant elevations in superoxide dismutase, glutathione peroxidase and lipid peroxidation. Notably, a significant decrease was observed in the glutathione levels after exposure to both copper forms. CuONPs only induced a significant increase in glutathione reductase and glutathione S-transferase. The ionic copper only induced a significant decrease in catalase activities in the gill tissues. Overall, CuONPs and Cu^2+^ provoked oxidative stress, and further research is needed to clarify their genotoxic and neurotoxic effects on freshwater mussels and other biota.

## Introduction

Nanotechnology is an evolving field focused on the research and development of nanoparticles with sizes less than 100 nm (NPs). Currently, engineered NPs are considered environmental pollutants because of the development of their applications in many research and industrial fields, such as food and food packaging, medicine, electronics, bioremediation, fuel catalysts, cosmetics, paints, coatings, and water treatment [1]. Alloys, metals, and carbon-based materials such as silicates and polymer products are considered engineered NPs [2].

NPs have unique characteristics that might increase chemical reactivity and biological activity due to their size. NPs can induce free radicals, resulting in severe toxicity in living organisms [3]. Moreover, NPs might induce negative effects on organisms via the penetration of their biological barriers and easy movement through the biological systems. NPs and their by-products are mostly released in aquatic ecosystems, showing their different effects on aquatic organisms [4].

Copper is an essential metal that acts as a cofactor in many biochemical enzymatic reactions, such as superoxide dismutase and cytochrome oxidase. Copper is a concentration-dependent metal; if found in higher concentrations within biological systems, it might induce organismal toxicity [5]. Copper oxide nanoparticles (CuONPs) are increasingly involved in several applications, such as wood preservation, bioactive coatings and liquid filtration in addition to electric coatings, due to their high conductivity [6]. CuONPs have pronounced bactericidal properties that result in their incorporation into inks, skin products and textiles [7,8]. Several test organisms, such as bacteria (*Escherichia coli*, *Bacillus subtilis*, and *Staphylococcus aureus*); protozoa (*Tetrahymena thermophila*); algae (*Pseudokirchneriella subcapitata*) and crustaceans (*Daphnia magna*, *Daphnia pulex*, and *Ceriodaphnia dubia*) in addition to zebrafish (*Danio rerio*) [9,10].

Invertebrates make up approximately 95% of all animals and play an important ecological function by transferring NPs within the food industry [11]. *Chambardia rubens* are native bivalves with rounded ventral margin and a shorter hinge line [12]. In Egypt, they inhabit the bottom of Nile from the south to the north. They represent a source of food and is widely used in the food chain [13]. In addition, they can be utilized for freshwater biomonitoring owing to their filtration ability and extensive geographical distribution [14]. The effect of copper accumulation in the tissues of this kind of bivalve is rare. Research on ’the biological consequences of both inorganic and organic pollution was achieved in invertebrates and particularly bivalve molluscs such as mussels [15–17]. Bivalves are filter-feeding molluscs that represent a major target category for the toxicity of nanoparticles because they establish endocytosis and phagocytosis processes for the cellular uptake of micro- and nanoscale particles, respectively [18]. In accordance with their filter feeding practices, the bivalve gill epithelium serves as the first interaction between the organism and the surrounding environment as well as the major route of exposure to environmental toxins. After exposure to NPs, gills are the first organ to be targeted via direct exposure or particle uptake [11]. In addition, digestive gland cells are the organism’s end destination for filtered particles; thus, the fate of NPs and their effects in mussels must be determined [19].

The digestive gland (hepatopancreas) of many molluscs is the major site of metabolism and is responsible for the production of digestive enzymes, the endocytosis of food substances, the absorption of nutrients, and for food storage and excretion [20]. In addition, after exposure to organic or inorganic pollutants, the hepatopancreas is considered to be the site of metal accumulation and cellular alterations [21,22]. The possible mechanisms of NP toxicity at the cellular level have not been completely identified, but they may include interruptions in energy transduction, genotoxicity, disruption of membranes, oxidation of proteins and formation of reactive oxygen species (ROS) [23]. In mammalian models, different experimental investigations have revealed the molecular mechanism of NP toxicity, including the production of oxidative stress through the release of highly reactive hydroxyl radicals [24,25], which also occurs in many aquatic organisms, including freshwater fish such as zebrafish [6] and mussels [26–28].

CuONPs showed toxicity to both vertebrates and invertebrates via free radical (ROS) release, which might result in the alteration of the antioxidant capacity by disturbing the activities of antioxidant enzymes [29,30]. Although many studies have used marine invertebrates to investigate NP toxicity [15,16,31], the possible impacts of CuONPs in freshwater bivalve species are poorly defined. To date, there are no comprehensive data, up to our knowledge, on the risks associated with heavy metals on freshwater mussels in Egypt. The current research was carried out to assess the toxic effects of CuONPs compared with ionic Cu^2+^ in both the gills and digestive glands of the freshwater mussel *Chambardia rubens* in this region. For this purpose, oxidative stress parameters were measured, including the activity of antioxidant enzymes [catalase (CAT), glutathione (GSH), glutathione peroxidase (GPx), glutathione reductase (GR), and superoxide dismutase (SOD)] in addition to lipid peroxidase (LPO). Furthermore, histological changes in the digestive gland and gills of this bivalve can be used successfully to monitor the effects of exposure to both copper forms.

## Materials and methods

### CuO nanoparticles (CuONP) synthesis and characterization

Copper nitrate and urea were used as the starting precursors in the preparation of CuONPs. Separate homogeneous solutions of copper nitrate (0.1 M) and urea (0.1 M) were prepared first using deionized water. A 50 ml aliquot of urea was added dropwise with a burette to 50 ml of copper nitrate solution using constant stirring at 80°C for 3 h. The precipitate was then separated via centrifugation at 10,000 × ***g*** for 15 min and properly washed three times with deionized water using sonication and centrifugation to remove the excess unreacted nitrate and urea. Then, the CuONP precipitate was left to dry in an oven at 100°C for 24 h and then annealed at 300°C for 4 h. The CuONPs were stored in closed bottles at room temperature for further experiments.

The average size and zeta potential for CuONPs dispersed in deionized water were analyzed at 25°C by dynamic light scattering (DLS) using a Malvern Zetasizer 2000 (Malvern, U.K.). Nanoparticles were suspended in deionized water by applying sonication before each measurement. Additionally, the shape and particle size of the CuONPs were examined by TEM analysis using a JEOL JEM-2100 electronic microscope (Ltd., Japan). The specimens for TEM measurements were prepared by depositing a droplet of CuONP suspension on a carbon-coated film (400 mesh) copper grid, after which the solvent was evaporated in the air at 25°C.

### Laboratory assay

*Chambardia rubens* mussels (75 ± 5.8 mm) were collected from the Banha region (AL-Qaluobiya Governorate) of Egypt. Then, they were exposed to steady temperature and aeration for one week for the acclimation process. The mussels were divided (approximately 2.5 mussels/L) into 20 L tanks filled with 15 L of fresh water in triplicate. The first three mussel tanks were exposed to 100 and 1000 µg Cu L^−1^ by adding CuONPs. The second three tanks were exposed to 100 and 1000 µg Cu L^−1^ by using CuSO_4_. The last three tanks served as controls. The copper exposure (CuONPs and Cu^2+^) was continued for 14 days, and the water was renewed every 12 h with fresh water that had been re-dosed with CuONPs and Cu stock solutions. CuONP solutions were sonicated for 30 min (45 kHz frequency) before each renewal to prevent aggregation. The control tank water was also renewed every 12 h. The mussels were not fed during the exposure, and no mortalities were observed. Cu-treat (CuONPs or Cu^2+^) and control mussels were collected after 3, 7, and 14 days, and then their biotic parameters were measured. The collected specimens were dissected; their digestive glands and gills were separated, immediately frozen and stored at −80°C until further analysis.

### Cu concentration in mussel tissues

A stock standard solution of Cu(II) ions at a concentration of 1000 μg/ml was prepared from copper(II) sulfate anhydrous (≥ 99.0) (Merck), and the working standard solutions were obtained daily by stepwise dilutions of the stock solution with doubly distilled water. The copper concentrations in the digestive gland and gill tissues were estimated using the method by Bagherian et al. [32]. Digestive gland and gill tissue samples were dried at 60°C, and combustion was performed at 450°C for one day. Then, the resulting samples were dissolved in a hot solution of 1 M HNO_3_. The digested samples were adjusted to 50 ml using deionized water in 50 ml volumetric flasks and then analyzed at 324.8 nm using a flame atomic absorption spectrophotometer (Perkin-Elmer, 3100). The absorbance of the sample solution was measured against the blank solution. The difference between the absorbance of the sample and blank solutions at 324.8 nm was used as an analytical signal. A calibration curve was constructed by plotting the analytical signal versus the Cu(II) concentration in a series of working standard solutions. The amounts of copper in the digestive glands and gills are presented as µg/g wet tissue weight.

### Oxidative stress and antioxidant biomarkers

The activities of antioxidant enzymes were determined in the digestive glands and gill cytosolic fraction of five mussels from the control and treated groups. The digestive glands and gills were immediately dissected and weighed. Ice-cold 50 mM Tris-HCl buffer (pH 7.4) homogenates (10% w/v) were prepared by mixing a part of the gills or digestive glands in ice-cold buffer using a tissue homogenizer and centrifuged at 3000 × ***g*** for 10 min at 4°C. The supernatants were collected and stored at −80°C for analysis. The homogenized digestive gland and gill total protein content was estimated according to Lowry et al. [33] using bovine serum albumin as a standard. The activities of several antioxidant enzymes, namely, catalase (CAT), glutathione peroxidase (GPx), superoxide dismutase (SOD), and glutathione reductase (GR), were estimated according to Sun et al. [34], Aebi [35], Paglia & Valentine [36], and Factor et al. [37], respectively. The levels of nonenzymatic antioxidant markers, such as glutathione (GSH), were measured according to Ellman [38]. Furthermore, based on Ohkawa et al. [39], the lipid peroxidation (LPO) was estimated using malondialdehyde (MDA).

### Histological examination

Based on Carleton et al., the mussel digestive glands and gills were removed and processed [40] and then fixed in 10% formalin for 12 h. Furthermore, they were dehydrated for 3 h at each copper concentration in an ascending series of ethanol concentrations (80%, 90%, and 100%), cleared in 2 changes of xylene (30 min each) and embedded in paraffin. Sections were cut by a microtome at a thickness of 5 μm, mounted on slides, dewaxed in xylene, stained with haematoxylin and eosin and then covered with glass slips using Canada balsam.

### Statistical analysis

The values are expressed as the means ± standard deviation (SD) of five mussels. The data were analysed using a one-way analysis of variance (ANOVA), and then, post-hoc Duncan multiple tests were performed. *P* values < 0.05 were considered statistically significant.

## Results

### Characterization of CuONPs

#### DLS for particle size and zeta potential analysis

**Figure 1:**
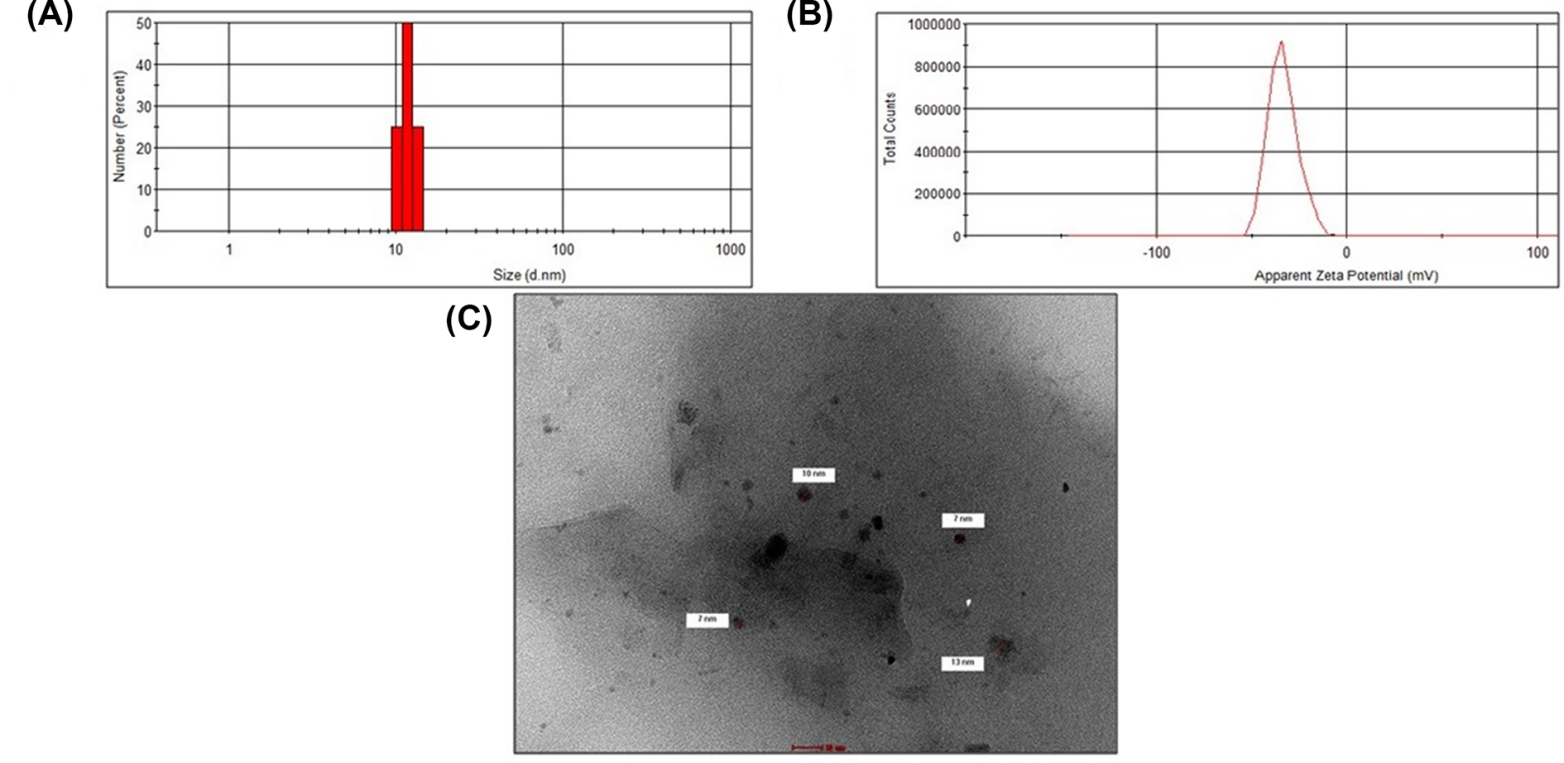
CuONPs characterization (**A**) DLS histogram, (**B**) Zeta potential, and (**C**) TEM micrograph.

DLS is an important and emerging technique for determining the particle sizes in a colloidal solution based on Brownian movement [41]. The size of NPs is one of the most critical properties for their applications. A DLS histogram of CuONPs is provided in Figure 1A. As shown in the figure, the average size of the CuONPs was 11.7 nm.

The zeta potential is a significant measurement for estimating the surface charge of nanoparticles, which is useful for determining their colloidal stability [42]. Hence, the surface charge of CuONPs was determined by zeta potential analysis and is presented in Figure 1B. CuONPs showed a mean zeta potential of −33.8 mV, indicating that they were stable.

#### TEM analysis of CuO NPs

The morphology of the prepared CuONPs was examined using TEM, and a typical image is depicted in Figure 1C. As indicated, the CuONPs are almost spherical, with an average size of 10 nm, which is consistent with the DLS analysis of their particle size.

### Copper content in mussel tissues

**Figure 2:**
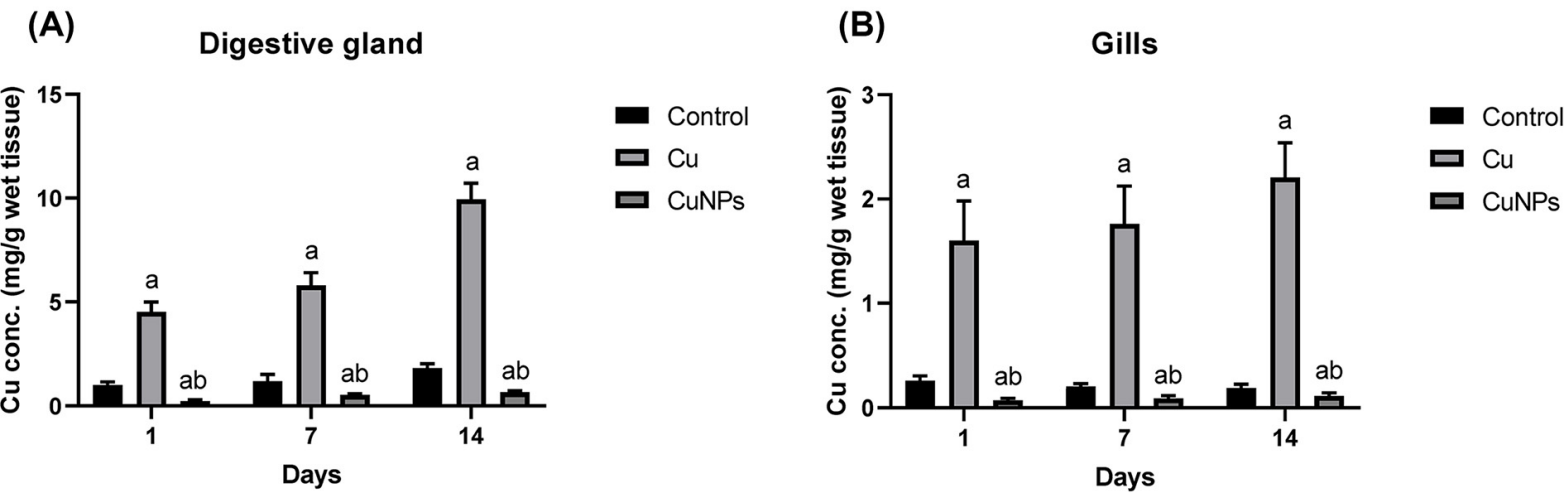
Cu concentrations (mg Cu g^−1^ wet tissue) in the studied tissues, digestive glands and gills (**A**) Cu concentration in mussel digestive glands after 3, 7, and 14 days of exposure, and (**B**) Cu concentration in mussel gills after 3, 7, and 14 days of exposure. Data are presented as the means and standard deviation. Significant differences between treated and untreated mussels are indicated with “a” at *P*<0.05. Significant differences between mussels treated with CuONPs and ionic copper are indicated with “b” at *P*<0.05.

Cu accumulation in different mussel tissues depends on the metal form and the period of exposure. After 3, 7, and 14 days of exposure, mussels treated with ionic copper displayed a significant increase in Cu content compared with NP-exposed and control mussels. The digestive gland accumulated higher levels of copper than the gills. Figure 2A,B.

### Biochemical results

**Figure 3:**
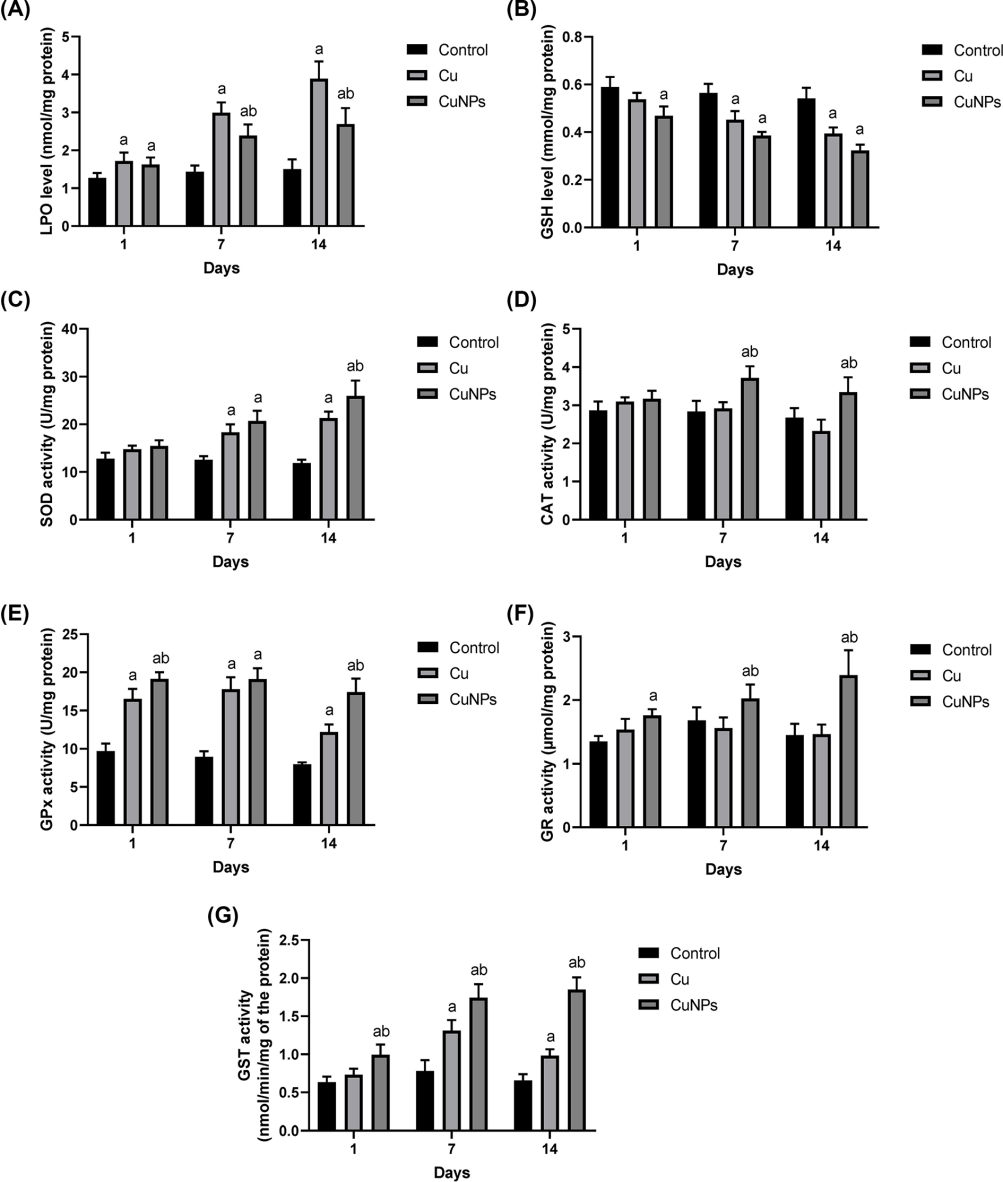
Oxidant/antioxidant status in the digestive glands of mussel *Chambardia rubens* following exposure to CuONPs and ionic copper for 3, 7 and 14 days (**A**) Lipid peroxidation (LPO), (**B**) glutathione (GSH), (**C**) superoxide dismutase (SOD), (**D**) catalase (CAT), (**E**) glutathione peroxidase (GPx), (**F**) glutathione reductase (GR), and (**G**) glutathione S-transferase (GST). The results are given as the means and standard deviation. Significant differences between treated and untreated mussels are indicated with “a” at *P*<0.05. Significant differences between mussels treated with CuONPs and ionic copper are indicated with “b” at *P*<0.05.

Different antioxidant enzymatic activities were found in the mussel digestive glands and gills after exposure to nanoparticles and ionic forms of copper. In the digestive glands, the LPO level in mussels exposed to CuONPs and ionic copper for 3, 7, and 14 days increased significantly in comparison with the control animals. The LPO levels were elevated over time in mussels treated with both forms of copper, as shown in Figure 3A. The GSH contents in mussels exposed for 3, 7, and 14 days to CuONPs and ionic copper decreased significantly in comparison with the control animals. The GSH content diminished over time in mussels treated with both forms of copper (Figure 3B). The SOD activity in mussels exposed CuONPs and ionic copper for 7 and 14 days to increased significantly in comparison with the control animals. The SOD enzymatic activity was elevated in mussels treated with both forms of copper, as shown in Figure 3C. The CAT activity increased significantly in mussels exposed for to CuONPs 7 and 14 days with respect to the control animals (Figure 3D). The GPx activity in mussels treated with CuONPs and ionic copper increased significantly with respect to the control after 3, 7, and 14 days of exposure (Figure 3E). GR activity in mussels treated with CuONPs increased significantly with respect to the control after 3, 7, and 14 days of exposure. In mussels treated with CuONPs and ionic copper, the GR activity increased over time (Figure 3F). The GST activity in mussels exposed to CuONPs for 7 and 14 days increased significantly in comparison with the control animals (Figure 3G).

**Figure 4:**
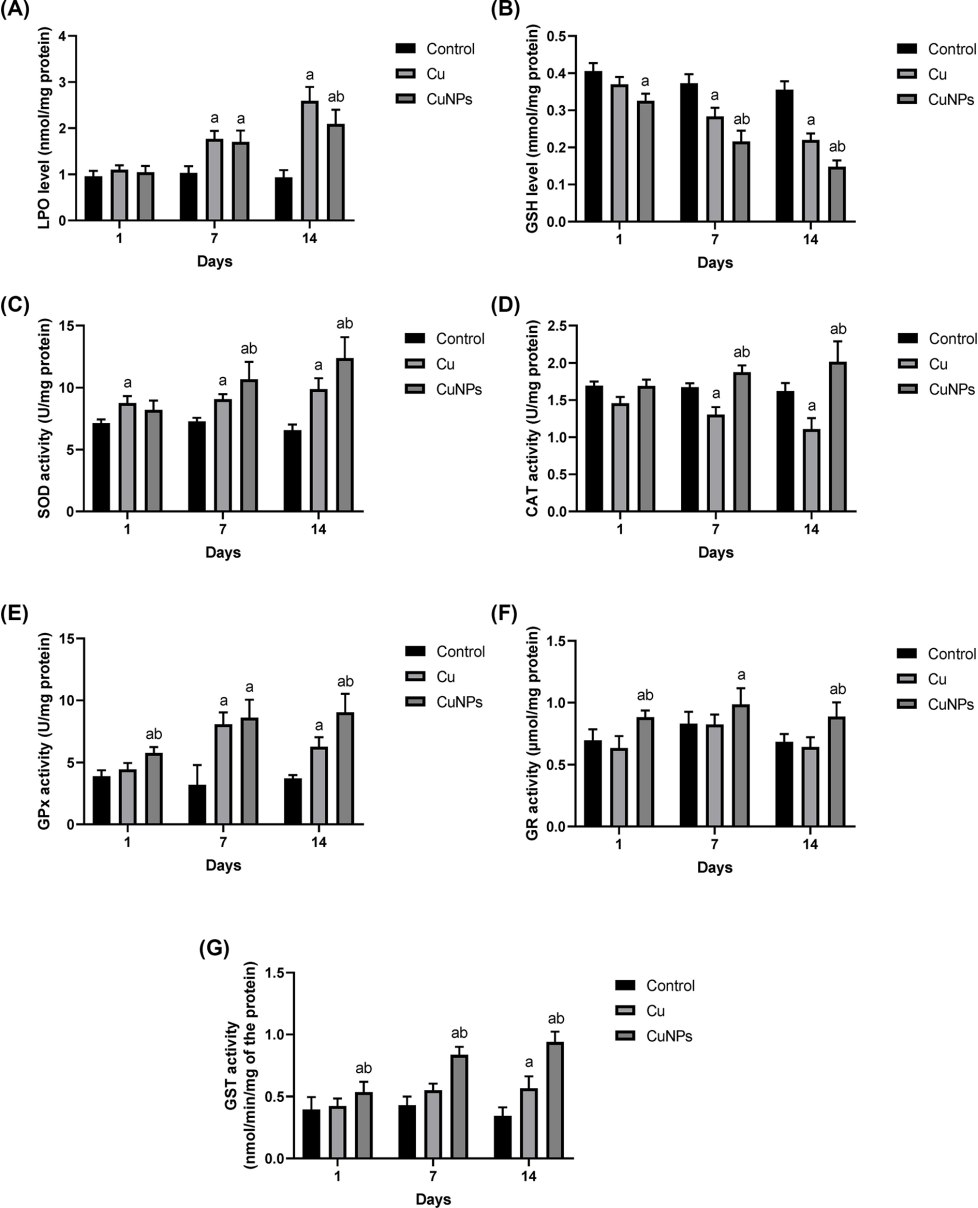
Oxidant/antioxidant status in the gills of mussel *Chambardia rubens* following exposure to CuONPs and ionic copper for 3, 7 and 14 days (**A**) Lipid peroxidation (LPO), (**B**) glutathione (GSH), (**C**) superoxide dismutase (SOD), (**D**) catalase (CAT), (**E**) glutathione peroxidase (GPx), (**F**) glutathione reductase (GR), and (**G**) glutathione S-transferase (GST). The results are given as the means and standard deviation. Significant differences between treated and untreated mussels are indicated with “a” at *P*<0.05. Significant differences between mussels treated with CuONPs and ionic copper are indicated with “b” at *P*<0.05.

In the gills, the LPO level in mussels exposed to CuONPs and ionic copper for 7 and 14 days increased significantly in comparison with the control animals. The LPO level increased over time in mussels treated with both forms of copper (Figure 4A). The SOD activity in mussels exposed to CuONPs and ionic copper for 7 and 14 days increased significantly in comparison with the control animals. The GSH content in mussels under the same conditions decreased significantly in comparison with the control animals. The GSH content decreased over time in mussels treated with both forms of copper (Figure 4B). The SOD activity increased over time in mussels treated with both forms of copper (Figure 4C). The CAT activity increased significantly in mussels exposed to CuONPs for 7 and 14 days with respect to the control animals, while it decreased significantly in mussels exposed to ionic copper for 7 and 14 days (Figure 4D). The GPx activity in mussels treated with CuONPs and ionic copper increased significantly with respect to the control after 7 and 14 days of exposure (Figure 4E). The GR activity in mussels treated with CuONPs increased significantly with respect to the control after 3, 7, and 14 days of exposure, while insignificant changes were noticed when they were treated with ionic copper (Figure 4F). The GST activity in mussels exposed to CuONPs for 3, 7, and 14 days increased significantly in comparison with the control animals, while it increased after 14 days of exposure to ionic copper (Figure 4G).

### Histological examination

**Figure 5:**
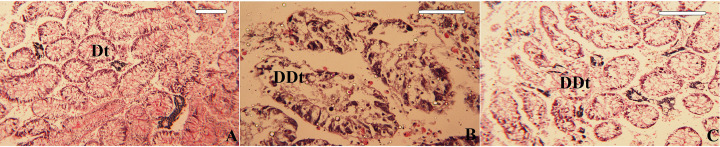
Photomicrograph of the *Chambardia rubens* digestive gland (**A**) Histological section of the digestive gland showing digestive tubules (Dt) of untreated (control) mussels, (**B**) Histological section of the digestive gland showing degenerated digestive tubules (DDt) of CuONP-treated mussels, and (**C**) Histological section of the digestive gland showing degenerated digestive tubules (DDt) of Cu^2+^-treated mussels. Magnification power is 100 µm.

The digestive glands of the control *C. rubens* mussels showed normal architecture, comprising a large number of digestive tubules. The tubules were isolated from each other by connective tissue. Columnar epithelial cells and secretory cells line each digestive tubule, which are supported by a basement membrane (Figure 5A). There were histopathological changes in their structure after exposure to 1 mg/L CuONPs and Cu^2+^, as observed in Figure 5B,C, respectively. The cells showed signs of necrosis and degeneration. In addition, many hepatic follicles showed overall damage.

**Figure 6:**
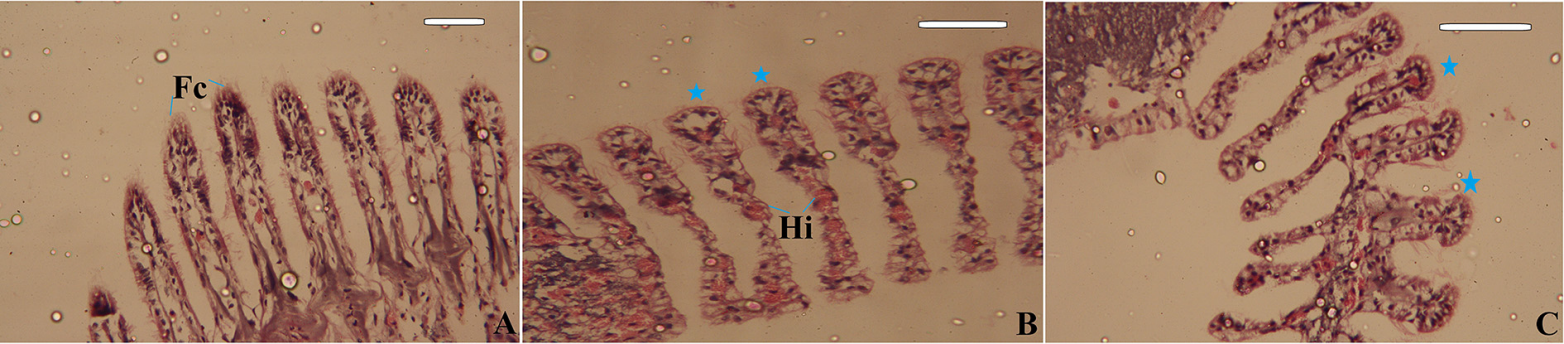
Photomicrograph of the gills of *Chambardia rubens* (**A**) Histological section of gills showing the presence of frontal cilia from untreated (control) mussels, (**B**) Histological section of the gills showing degenerated frontal cilia (arrows) and haemocytic infiltration (Hi) of CuONP-treated mussels, and (**C**) Histological section of the gills showing degenerated frontal cilia (arrows) of Cu^2+^-treated mussels. Magnification power is 100 µm.

Histological examination revealed normal gills in the control mussels (Figure 6A). The *C. rubens* gills consist of lamellae, filaments and water channels. Each gill filament was covered with frontal cilia. After exposure to 1 mg/L CuONPs and Cu^2+^, the frontal cilia of gill filaments degenerated, as observed in Figure 6B,C, respectively. Moreover, haemocytic infiltration was observed in the gill filaments of CuONP-exposed mussels.

## Discussion

Several studies have reported the toxicity of CuONPs in vertebrates and invertebrates [29,43–45], but the mechanisms of their toxicity have not been completely discovered. The toxicity of CuONPs could be explained by the release of toxic soluble copper from the NPs [10] or to the NP forms themselves [46]. However, Griffitt et al. [47] concluded that the dissolution of the NPs alone cannot explain the toxic effects that occurred in zebrafish after exposure to CuONPs, suggesting that they possess toxicity in a different way than soluble copper alone. Gomes et al. [48] confirmed Griffitt et al.'s [47] findings by determining the proteomic response of mussels after exposure to CuONPs and Cu^2+^.

In the present study, CuONPs and ionic copper were tested to determine the potential effects of CuONPs on the freshwater bivalve species *Chambardia rubens*. Exposing *C. rubens* to CuONPs for 14 days and to ionic copper induced Cu accumulation in the soft tissues of the mussels. The copper content was higher in mussels exposed to ionic copper than in those exposed to CuONPs in both digestive gland and gill tissues. Similarly, Ruiz et al. [49] found that the ionic copper concentration inside soft tissues was higher than that of CuONPs following exposure for 21 days. However, the copper concentration in mussels subjected to bulk CuO was less than that in the mussels subjected to CuONPs in the same study, implying that the bulk is less available than the CuONPs. Several creatures, such as mussels, have been observed to accumulate NPs at various speeds, with the highest concentrations in the digestive glands after exposure to specific NPs [30,50]. Moreover, in the present study, copper deposition in the digestive glands was two to four times higher than that in the gills for treated mussels, indicating that this tissue plays an important role in Cu bioaccumulation and detoxification [51]. Gomes et al. [48] found similar results after exposure of *Mytilus galloprovincialis* mussels to Ag nanoparticles, where this metal accumulated in the digestive gland 2- to 5-fold higher than that of the gills.

It is well known that increasing concentrations of ionic copper are highly toxic to aquatic organisms. Because copper is a transitional, redox-active metal, it participates in the Fenton and Habere-Weiss reactions, which promote the generation of reactive oxygen species (ROS) and lead to oxidative stress [52] that causes the activation/inhibition of several antioxidant enzymes [49,53]. In the present study, antioxidant enzyme activities in the *C. rubens* digestive gland and gills showed different responses after CuONPs and ionic Cu exposure, indicating the ability of both forms of copper to generate ROS and, notably, the effort of cells to compensate for the induced oxidative damage in these organs.

GSH is the most prevalent nonprotein thiol molecule in the body and plays a variety of critical physiological roles, including catalysis, metabolism, and transport [54]. Moreover, it acts as a principal cellular antioxidant in many tissues, safeguarding cells from free radicals, peroxides, and other harmful substances [55]. In the present study, GSH decreased after exposure to both CuONPs and ionic Cu for 7 and 14 days, while the GR and GPx concentrations were found to be constant during the same period of exposure. Similarly, Ali et al. [56] and Fahmy et al. [57] found that the GSH concentration decreases in the haemolymph, digestive gland and gills of *Coelatura aegyptiaca* and demonstrated that mollusc GSH concentrations drop as a result of metal exposure and that the metal has a strong affinity for the GSH molecule. The exposed sulfhydryl groups of GSH can bind to a number of electrophilic radicals and metabolites, potentially increasing cell sensitivity to a variety of toxic substances [58]. Therefore, the decrease in nonenzymatic antioxidants after Cu NP and ionic Cu exposure could be the consequence of increased action in trapping free radicals.

The GST enzyme participates in the detoxification process by facilitating glutathione conjugation with a variety of endogenous and xenobiotic compounds to form less toxic and more hydrophilic molecules [59]. This study revealed a significant increase in GST activities after CuONP and ionic Cu exposure in the digestive glands and gills of *C. rubens* compared with the control group. Our findings are similar to those of Buffet et al. [29], who found a significant elevation of GST activities in the soft tissues of *Scrobicularia plana* exposed to nanoparticles of copper oxide (CuONP) during 16 days of the experiment. Unlike the present study, Fahmy et al. [57] found a significant decrease in GST activity when *Coelatura aegyptiaca* was exposed to ZnNPs.

CAT and SOD are categorized as indispensable first-line defence antioxidants based upon their response to free radical invasion. In particular, superoxide anion radicals are generated in normal metabolism, primarily through the mitochondrial energy production pathway [60]. This study showed significant elevations in SOD activity in both tested tissues after exposure to CuONPs and ionic Cu for 7 and 14 days, whereas the CAT activity slightly increased or remained unchanged only in tissues exposed to CuONPs and decreased upon exposure to ionic Cu. The SOD activity increases following exposure to both CuONPs and ionic Cu, which could be attributed to their increased synthesis in response to oxidative stress. Therefore, this SOD activity increase might be a compensation for GSH depletion.

Notably, the subsequent higher levels in H_2_O_2_ from the SOD activity increase are not detoxified because of a decrease in CAT activity. The inhibition of CAT may occur either in response to an increase in the inhibitor superoxide anion or to a decrease in NADPH, since this coenzyme is required for full CAT activity [60]. Moreover, Fahmy and Sayed [61] linked a reduction in CAT activity in *C. aegyptiaca* mussels to a decreased ability to neutralize the ROS and a higher sensitivity to oxidative stress.

Gomes et al. [48] and Ruiz et al. [49] stated that the change in antioxidant activity relies on the Cu form and tissue type. In addition, Buffet et al. [29] found that after 16 days of CuONP exposure in *S. plana* mussels, the SOD activity (in all mussel bodies) was higher than that after ionic copper exposure. Similarly, Gomes et al. [53] studied the gills of *M. galloprovincialis* and found that CuONPs caused oxidative damage by exceeding the gill antioxidant defence system, but the enzymatic activity remained constant or increased following ionic copper exposure.

LPO is produced as a response to peroxidative damage by polyunsaturated fatty acids [54]. Increased LPO levels induce major implications, such as increased permeability due to membrane integrity loss resulting in a disturbance in the ion flow through the membrane, leading to dysregulation in the transportation of Na^+^/K^+^, high Ca ion flow and activation of catabolic enzymes such as proteases, phospholipases and nucleases [62]. In the present study, LPO activity showed a significant increase after exposure to CuONPs and ionic Cu for 3, 7, and 14 days for both study tissues, with a slight increase in the case of Cu ion-treated tissues observed at 7 and 14 days. Similarly, Bonnail et al. [62] found that the exposed Asian clam *Corbicula fluminea* induced slightly elevated levels of LPO in the presence of Cu >1 mg L^−1^ in water. Moreover, in Gomes et al. [53], the LPO increased linearly with time in *Mytilus galloprovincialis* mussels exposed to CuONPs and Cu^2+^ despite the efficiency of different antioxidants. In addition, exposure to CuONPs in human cells and *E. coli* led to lipid peroxidation, oxidative damage, and an increase in ROS, all contributing to oxidative stress [63]. In contrast, Goswami et al. [64] reported that nonenzymatic oxidative markers revealed no significant changes following Cu exposure in the marine green mussel *Perna viridis*.

The histological studies on the digestive glands and gills of *C. rubens* are similar to many reports on different bivalve species [63,65,66]. Moreover, multiple oxidative reactions may occur in the digestive gland and, therefore, could be a significant site of free radical release. Regoli et al. [67] reported that haemocytes transport pollutants to the digestive gland. Histological alterations of bivalve gill tissues have been shown to play a crucial role in food collection and respiration [68]. Therefore, the digestive gland and gills were employed in this research to evaluate the histological alterations of *C. rubens* to CuONPs and Cu^2+^.

In the present study, the atrophy of the digestive tubules and the changes in the morphology of the digestive tubules were observed histopathologically and are considered nonadaptive responses to pollution exposure [69]. Furthermore, alterations in the morphology of the digestive gland structure may be linked to other environmental elements, such as food availability, salinity, and heat stress, resulting in the failure of an organism’s digestion and storage activities as well as physiological impairment [70]. Similarly, Ruiz et al. [49] found haemocytic infiltrations in the gill filament of *Mytillus galloprovincialis* mussel following exposure to CuONPs. Hemocytic infiltration may represent a repair process following tissue damage [71]. Unlike our study, Ruiz et al. [49] discovered that brown cell aggregations increased over time, peaking at 122 days following exposure.

## Conclusion

Up to our knowledge, this is the first study to elucidate the toxic effects of copper along with its nanoform (Cu ions and CuONPs) on the bivalve *Chambardia rubens*. Both forms of copper were able to induce oxidative stress in the mussel digestive glands and gills. The distinct efficiency of enzymatic and nonenzymatic antioxidants and LPO in mussel tissues is dependent on the used Cu form and the exposure time. Digestive gland of the bivalve *C. rubens* showed more sensitivity to the oxidative stress than gills tissues for both copper forms and it was the main target tissue for Cu accumulation and pronounced histopathological alterations. In the future studies, CuONPs toxicity requires more investigation to determine if the observed toxic effects are primarily due to free Cu ions dissociated from the NPs or to the mixture of the NPs’ effects.

## Data Availability

We confirm that all original raw data is available at the time of submission. As per the Data Policy, these data will be stored for a minimum of 10 years and will be made available to the Editorial Office, Editors and readers upon request.

## Abbreviations

CAT: catalase
DDt: degenerated digestive tubules
GPx: glutathione peroxidase
GSH: glutathione
LPO: lipid peroxidase
ROS: reactive oxygen species
SOD: superoxide dismutase
